# Variance in multiplex suspension array assays: carryover of microspheres between sample wells

**DOI:** 10.1186/1477-5751-6-6

**Published:** 2007-04-25

**Authors:** Brian Hanley

**Affiliations:** 1 Microbiology Graduate Group, University of California, Davis, CA 95616, USA; 2 BW Education and Forensics, 2710 Thomes Avenue, Cheyenne, Wyoming 82001, USA

## Abstract

**Background:**

This study was undertaken because of the accidental observation that a sample of 60+ beads was obtained by the instrument from a completely dry, unused well in a 96 well plate. Others have observed unexplained outliers in replicated wells. The problem was first observed on an older instrument, and replicated on a new instrument.

**Methods and results:**

Data is presented from two instruments using a multiple blank following well experiment that shows a surprising amount of carryover that has an unexpected nature. When it occurs, it does not necessarily decline from one well to the next. There appears to be two types of carryover, one that is small, predictable and declines consistently, and another which is potentially very large, unpredictable, and does not decline. The former can be compensated for or ignored. The latter cannot be addressed without using multiple replicated samples or an intraplex method.

**Conclusion:**

This problem has significance for analysis of results obtained with suspended microarray instruments. A special notation is made that biostatisticians need to be made aware of these results before experiments are undertaken and data generated for them to analyze. The problem can be handled by enough replicated samples, or an intraplex method. The applicability of these results to oligonucleotide based assays is unknown.

## Background

A suspended microarray assay system uses small particles such as microrods or microbeads that contain some method for identifying a set. An assay used to detect an analyte is bound to the surface of a set of identical particles, which are generally in the size range 3–15 microns. These particles are added to a liquid containing the analyte. (In systems such as "smart dust", the assay may be distributed in the field to detect analytes.) The final step in the assay activates a fluorophore that provides a signal. The particles are run through a flow cytometer, which is generally optimized for the specific system used. For each particle in the mixture, the cytometer identifies the classifier together with the fluorescence reading of the reporter fluorophore. Because the particle classifiers are unique for each analyte, it is possible to multiplex the assays together in a test tube. Alternatively, multi-well assay plates can be used, and such assays then become a high throughput system.

The Luminex assays compared in this study utilize microbeads on which antigens or antibodies have been covalently bonded (xMap™ assays). xMap™ microbeads contain two reporter fluorophores, which are proportionally varied to identify them as one of 100 possible bead identifiers. Classical sandwich assays such as streptavidin-linked phycoeryrthrin are conducted to attach reporter molecules to the beads. The reporter fluorophore intensity is then measured in a specialized flow cytometer together with the microbead identifiers and the fluorescence measurement is classified by bead identifier. A sample of *n *beads is collected, and median, mean, or trimmed mean are generally used as the reported value. The system is typically deployed with one well, or sometimes two wells containing the same analyte fluid.

The fluid with a sample of microbeads flows up through a probe, which has a tip with 5 very fine holes leading to a single channel at the top. The fluid travels through a system of tubing and valves into the flow cell, where (in the current equipment) two lasers are present. One laser stimulates the two marker fluorophores, and the other stimulates the reporter fluorophore. A system of avalanche photodiodes and photomultiplier tube captures and reads the fluorescence from marker and reporter emissions [1,2].

The usual number of beads that are recovered and used by the instrument is 50 to 100 per bead set. Assays with counts as low as 30–35 are used. In separate experiments (not shown) using 32 replicates at varying bead counts, no significant difference in replicate standard deviation was seen until 700 to 1,000 beads are counted. The improvement at higher counts was minor.

Assays are normally done putting 1,000 to 2,000 beads per bead type into one well. This is the number necessary in order to acquire 35–100 beads at the end of the assay. Higher bead counts require proportionally higher doses of beads for the assay. It takes a long time to acquire large numbers of beads if it works. It can take so long that the instrument becomes impractical to use for its purpose of high throughput. Additionally, the beads are precision manufactured product, and expensive.

For a diagnostic test, these assays have a cutoff value established by the assay designer. If an assay goes over that value, it is positive, if it is under the cutoff value it is negative.

The assays used for this study were all protein antigen/antibody assays. The instruments in use for these assays were instruments that had run such protein antigen/antibody assays. There are plausible reasons to question if these results apply to deoxyribonucleotide based assays; this is addressed as part of the discussion. Deoxyribonucleotide experiments were not possible within the scope of this study.

### Design considerations of experiment

The experiment was designed as one of a set undertaken to tease apart the various contributors to variance in the Luminex assay system. There are more than a few contributors to variance, (on the order of at least 10) so the question was how to isolate the contribution of carryover between wells. In a normal assay, in which each well is filled with biological material to be analyzed and fluorescence is read, it would be impossible to point to a fluorescence result and say that it was specifically due to carryover because of the large number of other sources of variation in the system including stochastics. Consequently, the experiment was designed to only count beads in each well, and nothing else. Bead counts are supplied by the instrument. Those counts are then used proportionally to project how they could change an assay.

The original observation that prompted prioritizing a carryover experiment occurred in dry wells. Thus, one early idea for an experiment was to put a dry plate into the instrument and run it through for 96 wells. However, that would not be normal operation of the instrument. Such conditions would create cavitation and bubbles inside the tubing and probe tip. It could be argued that while some amount of binding and release of beads might be occurring under normal conditions, the numbers would be insignificantly small compared to the scouring effect of cavitation. So this alternative was rejected as providing invalid results.

To identify beads that were carried over from prior wells, a plate was defined with rows A and E containing real sample and beads. After each well containing sample and beads were three empty wells. Therefore, if a bead was to appear in one of the three empty wells, it would have to be from carryover of some kind as long as there was a way to eliminate accidental contamination.

A set of preliminary experiments were conducted where 4 technicians in the lab pipetted 3 μl into wells. Preliminary to that, the rate of evaporation at various locations on lab benches were assayed using a high sensitivity scale. These tests required exact timing of pipetting and weighing since evaporation occurs rather quickly. The results showed that at 3 microliter quantities, large variances occurred. Some wells had double inoculations. These results indicated that great care and some type of double-check had to be in place against bench error to accept any results.

Consequently, it was decided that unless the intervening wells were dry until the last step, just prior to going into the Luminex instrument, the experiment would not be valid. This is the best method of visual control to ensure visibility of injection of beads into a well by accident. All beads are injected in suspension, so the liquid would show in the well as a different color. Dyes were rejected since in the lab they were not normally used, so their potential change in effect on any assay was unknown.

Additionally, since the wells are washed multiple times during the assay, there is no biological sample during the assay in the follow-on empty wells that could affect any result.

For those not familiar, in outline, the way these assays are conducted is as follows:

1) Sample (lysate, serum, etc) is pipetted in dilute form into wells.

2) A mixture of different beads are injected into wells. *Note: Typical bead counts are ~1,000 to 2,000 beads per well for each bead type*.

3) Plate is incubated. This ranges from 2 hours to overnight depending on sample and assay.

4) Plate is washed twice times with PBS Tween.

5) The second antibody is pipetted into the wells and incubated. Again, timing can vary on incubation time.

6) Plate is washed 3 times with PBS Tween.

7) Phycoeryrthrin (or another reporter) is pipetted into wells. This time incubation is for 30 minutes so that all assays have roughly the same amount of phycoerythrin bound to reporter antibodies.

8) Plate is put on the Luminex assay platform and assayed.

The outcome of these experimental design considerations is that the impact of bead counts on fluorescence results must be made by deduction as a general principle. By observing counts, and applying known principles, the potential effect on assays is made clear. The import of this experiment can only be general, it cannot be made specific for a well in a real assay by any conceivable method.

## Methods

The assays used in this study were developed previously for a simian virus detection project. They were manufactured using carboxylate xMap™ microspheres from Luminex (Luminex; Austin, TX) conjugated to viral antigens; the viral antigens are identified in the appendix together with the bead classifiers. The assays were antigen attached to microspheres, intended to bind Rhesus macaque antibody.

Uncoated beads were used as controls, together with microbead assays for which the serum sample was known to be negative. Frozen serum from a single Rhesus macaque with known positive and negative characteristics was used as the sole experimental sample (see Appendix). Samples were incubated for two hours on a shaker table, washed, then incubated for 40 minutes with R-Phycoerythrin-conjugated Affinipure F(ab) Fragment Goat anti-Human IgG Fcγ (Jackson ImmunoResearch Laboratories, Inc.; West Grove, PA), which was used as a conjugate reporter to detect the Rhesus macaque antibodies bound to beads. The plate contents were then washed, shaken to suspend the microbeads, washed again, resuspended, then read on a Luminex instrument. Plates were stored overnight a 4°C refrigerator and read on a Bioplex instrument the following morning.

### Preparation of xMap™ microspheres

Details of the bead preparation are given in the appendix (xMap™ bead coating protocol.) The use of beads with recorded assay results was accepted as sufficient indication that they were representative of a real world assay, which was the objective, even though bead counts was the only data used. The assays used in this study were antigen attached to microspheres, intended to have Rhesus Macaque antibody bind to the antigen. R-Phycoerythrin-conjugated Affinipure F(ab) Fragment Goat anti-Human IgG Fcγ (Jackson ImmunoResearch Laboratories, Inc.; West Grove, PA) was used as conjugate reporter to the Rhesus macaque antibodies bound to beads.

In Table 1 are listed the virus antigens used in these experiments, with bead identifiers. A 100 s digit was prefixed to differentiate in-house assays from those acquired from outside (106 = bead region 006, 112 = bead region 012, etc.)

**Table 1:** Assays and bead classifiers available for use

**CMV**	**SFV**	**SRV**	**SIV**
106	105	**146**	104
112	111	147	133
113	115	152	137
180	118	197	
	**166**	198	
	173		

CMV = Cytomegalovirus

SFV = Simian Foamy Virus

SRV = Simian Type D Retrovirus

SIV = Simian Immunodeficiency Virus

Items in bold are duplicated bead identifiers which were not used.

### Preparation of microtiter plates

The plates used were MultiScreen HTS, BV (Millipore; Bedford, MA) 96 well filter plates. Preliminary studies of pipetting error indicated that volumes above 5 μl would have minimal error. All assays were conducted such that no fluid volume below 5 μl would be pipetted. On the basis of preliminary evaporation studies, a total volume of at least 90 μl per well was used during incubations to minimize evaporation as a source of variance. In addition, all wells were filled within 10 minutes or less so that any difference between well concentrations due to evaporation was further minimized.

### Experiments

Protocol 3 – Samples were laid out in rows A and E across the plate, and all other wells were left dry during incubation steps. Straight PBS-Tween was added to all wells during the final suspension step to preserve normal fluidic operation of the Luminex instrument. This made any microbeads that might appear in a following empty well attributable to something other than the well itself.

### Data collection

For these experiments two instruments were used. One is a Luminex model 100 that is approximately 5 years old. The other is a Biorad Bioplex instrument that was installed in late December 2005 and was commissioned for use in January of 2006. Both instruments were under standard service contract. Prior to commencing the study, both instruments had been serviced by field technicians in the previous 2 months. Also prior to commencing the study, the older Luminex instrument was upgraded to the latest software and firmware levels. The only data used for this experiment was the count of beads for each well.

## Discussion

On one plate, 2 dry wells, F7 and F8 were observed to have a bubble of fluid on them after incubation. It was presumed this was from some action of the shaker table, the plate lid and evaporation/condensation. However, that plate showed no more carryover of beads for those two wells than for any other.

In summary, the results show that carryover between wells can vary a great deal. The factors contributing to the carryover that are seen in this experiment are unknown. However certain factors such as probability of accidental deposition of microbeads into wells showing anomalously high carryover can be ruled out. Empty wells were left dry deliberately so that any such accidental deposition would be visible. Additionally, those wells which showed the highest anomalous values were at the bottom edge of the plate as shown below. They were not neighbors of wells with sample. An example of this is shown in Table 2 for one bead identifier. The values are counts of beads. Rows A and E in bold italics had wells containing beads. Rows B, C, D and F, G, H, in normal text were empty of samples, and filled with PBS-Tween solution just prior to reading. Upper left corner cell contains bead identifier.

**Table 2:** 

**125**	*1*	*2*	*3*	*4*	*5*	*6*	*7*	*8*	*9*	*10*	*11*	*12*
***A***	***267***	***394***	***370***	***404***	***347***	***381***	***348***	***91***	***407***	***315***	***370***	***347***
*B*	3	5	6	9	6	5	4	0	5	6	4	6
*C*	12	1	1	7	0	6	0	29	1	3	3	8
*D*	6	6	2	1	0	9	0	1	24	14	31	15
***E***	***314***	***342***	***354***	***322***	***307***	***350***	***290***	***212***	***226***	***185***	***21***	***19***
*F*	3	8	1	5	2	4	2	1	6	1	6	4
*G*	1	5	5	2	9	1	0	39	57	36	37	52
*H*	23	9	1	0	8	8	114	79	1	62	1	22

(Note, the bead number above was 25. Intra-lab assays have a preceding digit "1", hence 125.)

Data in bold italics is the row with actual sample. All other rows were empty, filled with buffer just prior to reading.

**Figure 1:**
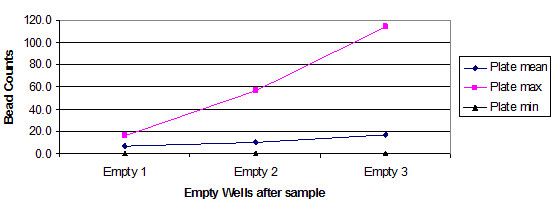
Summary of 6 bead sets, trailing empty well contents for instrument B. Bead counts.

**Figure 2:**
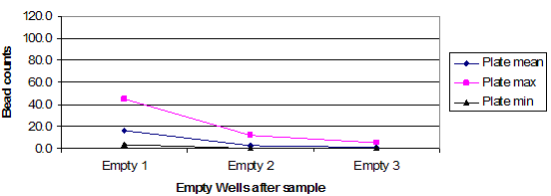
Summary of 6 bead sets, trailing empty well contents for instrument A. Bead counts.

The charts in Figure 1 and Figure 2 summarize the observations from these two plates. Instrument manufacturers are hidden because there is believed to be no significance to the vendor name for these results. One plate had some very high outliers, and as a consequence, the mean average and the maximum are an inverse of what one would expect, with rising numbers rather than declining numbers. The primary point is that this could occur on any plate of samples and there is no way to know without running some form of replication.

### Carryover effect on fluorescence readings

There are two things that matter here for projecting range of effect of carryover on fluorescence reading. First is what percentage of the total number of beads acquired this carryover percentage represents. Second is what the absolute number of beads is that is acquired by the instrument attributable to carryover. This experiment puts a stake in the ground for both.

If one predicates that ~10% of the beads from a well are from the assay of a different well(s), and those beads are positive, then in any case where the true value would have been 10% or less *below *the cutoff value for positive diagnosis, the result is a false positive. Conversely, any case where the true value is 10% or more above the cutoff, and those beads from other wells are negative, then it will give a false negative diagnosis. Whether the beads from other wells will be positive or negative depends on the mix of samples. In many instances, most samples are negative. The data above supports a minimum possible carryover level of 10%.

Looking at the problem by absolute count, if one says that the maximum number of carryover beads is 114 as observed in this experiment, and compares that to the usual range of 50 to 100 beads acquired for a sample, then one can say that it is possible for any amount of sample, up to and including 90% or more of the beads acquired to be from a different well than that reported.

If one makes the assumption that the beads are first injected into the well and thoroughly mixed with those beads in the well, then one can assume that the above percentage rules should apply, subject to stochastic variations. With stochastic variations, there are "lottery winners" sooner or later; for patient diagnostics this would matter, since the diagnosis would probably be changed to the opposite of what it should be.

The problem is that the preceding assumption cannot be depended on. Since beads are by definition carried over from inside the instrument somewhere, at least some of the time a slug of beads could come loose that are carryover beads during the suction cycle into the instrument, and carryover beads precede those coming from the well. In such a case these carryover beads would be read first, followed by the correct beads. If a full set of 50 to 100 beads was acquired by the instrument, whether more beads were counted would depend on whether other bead sets had reached the lower limit cutoff value yet. The point here being that bead counts for a specific identifier would not necessarily be subject to dilution and mixing within the well. The degree of dilution of this slug of carryover beads could be as low as 10% or less.

What this indicates is that carryover from one well to the next is a significant issue as an unpredictable factor contributing to fluorescent intensity readings.

### Possible explanations for carryover phenomenon

No definitive explanations for the carryover phenomenon is presented here, but several possibilities are suggested: A.) Random differences in fluid adhering to the probe tip as it moves from well to well. B.) Small scratches or imperfections on the surface of the sampling probe may carry fluid. C.) Inside the probe, one or more of the fine channels may become temporarily blocked or occluded with a combination of materials and intermittently clear. Some candidate materials are: C.1.) Fibrinogen C.2.) Microbeads C.3.) Bacterial or fungal growth. D.) Adhesion and release could occur from valves and tubing internal to the instrument.

It is noteworthy that clogging of the probe tip is known to be a fairly common occurrence as evidenced by procedures provided by the instrument vendors for clearing clogged probes. Since there are 5 small holes in the probe tip, when a user realizes that the tip is clogged, this means that the tip has probably got three or less holes that work. There should also be velocity and fluid flow changes internally to the probe as holes clog, leading to unknown opportunities for deposition and adherence of microbeads in eddies on the fluid flow. It is also virtually guaranteed that any probe tip will have small differences in flow due to minor manufacturing imperfections. When a probe tip channel gets clogged, it will no longer have fluid flow (such as bleach or alcohol disinfectant), so bacterial and fungal growth is a virtual certainty to occur in the clogged channel. The clog will contain microbeads that could either potentially carry over to be deposited in another well, or else come loose and flow through into the instrument flow cell during an uptake cycle. The number of beads could be quite high potentially.

It also makes sense that the plumbing of an instrument would eventually support bacterial and fungal colonies despite flushing protocols, and that wear in valves and turbulence at fittings would be expected to create opportunities for adhesion, and "adhesion and release" of microbeads. This assumes that there would be no possible surfaces to which beads, fungi and bacteria could be expected to stick when the instrument is new, which is unlikely.

Antibody and antigen coated beads complexed with streptavidin-phycoerythrin reporter present ample chemistry for binding to surfaces such as steel and plastics. They also provide nutrition for microorganisms in the form of amino acids. Myxococcus xanthus, for instance, which is a common environmental bacteria, prefers amino acids. The attendant products of colonization by bacteria and fungi would additionally create more chemistry for binding and aggregation.

### Application of these results to deoxyribonucleotide bead assays

Whether nucleotide beads would exhibit the same behavior in an instrument used exclusively for nucleotide bead assays is not established by this experiment. For nucleotide based assays, nucleotides are a much poorer nutritional source than protein based assays. There are reports of facultative capacity to break down purines by some bacteria. However, energy yield is low, making this source unlikely to support significant growth. Additionally, nucleotides bind poorly to most plastics and steel.

## Conclusion

In antibody/antigen assays, carryover can occur that is significant enough to go over or under a cutoff value established for a diagnostic, and thus deliver an incorrect value. This carryover is not predictable in a manner that can be compensated for without replicates or intraplex assay design. In addition, the manufacturer provides remediation procedures for clogging of probes. Together, at minimum, these indicate that beads can clog in the tip and be released later, although whether the probe tip is the only location is not established. The manufacturer should study the problem of carryover and take steps to alleviate it, or else provide guidelines for use of assay methods that are robust enough to be able to compensate for it.

## Appendix

### xMap™ bead coating protocol

1. Vortex the uncoated beads for 20 seconds and sonicate for 1 minute.

2. Remove 250 μl (2.5×10E6) uncoated beads and put into a fresh 1.5 ml tube.

3. Spin the beads at 21000 × g 2 minutes.

4. Aspirate most of the supernatant without disturbing or drawing up the beads.

5. Pellet as many times as needed to remove supernatant without disturbing the beads.

6. Vortex the pellet

7. During the final spin, measure out Sulfo-NHS and EDC and dilute to 50 mg/ml. Once resuspended, the reagents must be used within 10 minutes.

8. Add 80 μl chilled Monobasic Sodium Phosphage, pH 6.3

9. Add 10 μl 50 mg/ml Sulfo-NHS to the microspheres.

10. Vortex.

11. Add 10 μl 50 mg/ml EDC to the microspheres

12. Vortex

13. Incubate on plate shaker 140 rpm 20 minutes at room temperature in the dark.

14. During the incubation take the prepared antigen and dilute it with MES (50 mM pH 6.0)

15. Centrifuge beads 21000 × g 2 minutes

16. Discard supernatant and vortex the pellet

17. Wash with 250 μl MES (must use MES to wash or coating will not work)

18. Repeat step 14–16

19. Pull the supernatant off of the second wash. Vortex the bead pellet

20. Add the 250 μl prepared antigen made in step 17 to the beads.

21. Vortex.

22. Incubate at room temperature in the dark for 2 hours on rotator.

23. Centrifuge 21000 × g 2 minutes. Pull the supernatant and vortex the pellet

24. Wash with 250 μl PBS-Tween20

25. Repeat steps 22–23 one more time

26. Centrifuge 21000 × g 2 minutes and pull supernatant. Vortex the pellet

27. Resuspend in 250 μl PBS-TBN for blocking

28. Incubate by rotation 30 minutes in the dark at room temperature.

29. Centrifuge 21000 × g 2 minutes, pull supernatant, vortex the pellet and resuspend in 1 ml PBS-TBN.

30. Count beads by diluting 1:50 (10 μl beads in 490 μl PBS/Tw) and running 100 μl in three wells. Average the bead count.

### Experiments

#### Summary

Use single monkey serum at the same dilution in each well using multiple bead sets detecting the same antigens. A set of at plates of identical sera with several identical assays was done against 32 wells × 3 assays per plate.

#### Prior protocol for all

1. Deactivate 3 ml of monkey serum at 56 C for 30 minutes in BSL-2.

2. Aliquot to 0.5 ml per tube. Refreeze. Intention is to remove number of freezings of sera as a variable.

Serum used: 1.5 milliliters of serum from monkey #26082.

This monkey is known positive for:

• SRV, CMV and SFV

Known to be negative for:

• SIV, STLV, HPV2

Serum was deactivated on 05/27/2006.

One freeze/thaw cycle occurred for all sera in study.

The tests were executed on both Luminex in the CCM (Center for Comparative Medicine) and the Bioplex machine at CNPRC (California National Primate Research Center).

### Protocol-3: 2 plates

#### Rationale

Previous pre-trial has shown that some bead counts cross contaminate from well to well at up to 4 wells beyond last well containing sample and beads.

#### Purposes

• Determine how many beads contaminate from well to well in machine.

• Variance of cross contamination.

N

• N = 24 per plate × 2 plates = 48

1. Prepare 2 chilled plates with 70 μl chilled PBS-Tween.

2. Prepare 1 dilution of monkey serum in Prionex,.

a. 1:100

3. Prepare enough of each dilution to have 50 μl of dilute sera per well for a final concentration of 1/2 the pre-plate concentration.

4. Put each dilution in row A and row E of each plate.

5. Fill rows B, C, D and F, G, H with PBS.

6. Beads are only placed in rows A and E.

7. Place bead mix composed of one of each of the below:

19 uncoated

22 uncoated

25 uncoated

32 uncoated

41 uncoated

89 uncoated

173 SFV 12.5 ug/ml

into each well.

8. Standard protocol for incubation, washing, and PE placement.
